# Comparing loss of p16 and MTAP expression in detecting *CDKN2A* homozygous deletion in pleomorphic xanthoastrocytoma

**DOI:** 10.1093/jnen/nlae076

**Published:** 2024-07-23

**Authors:** M Adelita Vizcaino, Caterina Giannini, Rachael A Vaubel, Aivi T Nguyen, Jorge A Trejo-Lopez, Aditya Raghunathan, Sarah M Jenkins, Robert B Jenkins, Cinthya J Zepeda Mendoza

**Affiliations:** Department of Laboratory of Medicine and Pathology, Mayo Clinic, Rochester, MN, United States; Department of Laboratory of Medicine and Pathology, Mayo Clinic, Rochester, MN, United States; Department of Biomedical and Neuromotor Sciences, University of Bologna, Bologna, Italy; Department of Laboratory of Medicine and Pathology, Mayo Clinic, Rochester, MN, United States; Department of Laboratory of Medicine and Pathology, Mayo Clinic, Rochester, MN, United States; Department of Laboratory of Medicine and Pathology, Mayo Clinic, Rochester, MN, United States; Department of Laboratory of Medicine and Pathology, Mayo Clinic, Rochester, MN, United States; Department of Quantitative Health Sciences, Mayo Clinic, Rochester, MN, United States; Department of Laboratory of Medicine and Pathology, Mayo Clinic, Rochester, MN, United States; Department of Laboratory of Medicine and Pathology, Mayo Clinic, Rochester, MN, United States

**Keywords:** CDKN2A homozygous deletion, chromosomal copy number variation, Immunohistochemistry, MTAP, p16, pleomorphic xanthoastrocytoma

## Abstract

Pleomorphic xanthoastrocytomas (PXAs) harbor *CDKN2A* homozygous deletion in >90% of cases, resulting in loss of p16 expression by immunohistochemistry. Considering the proximity of *MTAP* to *CDKN2A* and their frequent concurrent deletions, loss of MTAP expression may be a surrogate for *CDKN2A* homozygous deletion. We evaluated p16 and MTAP expression in 38 patient PXAs (CNS WHO grade 2: *n* = 23, 60.5%; grade 3: *n* = 15, 39.5%) with available chromosomal microarray data to determine whether MTAP can be utilized independently or in combination with p16 to predict *CDKN2A* status. *CDKN2A*, *CDKN2B*, and *MTAP* homozygous deletion were present in 37 (97.4%), 36 (94.7%), and 25 (65.8%) cases, respectively. Expression of p16 was lost in 35 (92.1%) cases, equivocal in one (2.6%), and failed in 2 (5.3%), while MTAP expression was lost in 27 (71.1%) cases, retained in 10 (26.3%), and equivocal in one (2.6%). This yielded a sensitivity of 94.6% for p16 and 73.0% for MTAP in detecting *CDKN2A* homozygous deletion through immunohistochemistry. MTAP expression was lost in the 2 cases with failed p16 staining (combined sensitivity of 100%). Our findings demonstrate that combined p16 and MTAP immunostains correctly detect *CDKN2A* homozygous deletion in PXA, while MTAP expression alone shows reduced sensitivity.

## INTRODUCTION

Pleomorphic xanthoastrocytoma (PXA) is a rare circumscribed glioma that predominantly affects pediatric and young adult patients.^1^ It often arises in superficial locations involving the leptomeninges, most commonly in the temporal lobe. Histologically, PXAs are characterized by a mixture of spindled, large pleomorphic and lipidized (xanthomatous) cells, pale and bright eosinophilic granular bodies, perivascular lymphocytic inflammation, and a rich pericellular reticulin network.^2^ In the current World Health Organization (WHO) Classification of Central Nervous System (CNS) Tumors, PXAs with low mitotic activity (<5 mitoses per 10 high-power fields [HPF]) are designated as grade 2, while PXAs showing brisk mitotic activity (≥5 mitoses per 10 HPF) correspond to a grade 3 (A-PXA) designation.^1^^,^^3^^,^^4^ PXAs harbor frequent MAPK pathway genetic alterations, with *BRAF* p. V600E mutation present in up to 78% of cases.^5^^,^^6^ In addition, homozygous deletion (HD) of the *CDKN2A* gene, located on the 9p21 chromosome region, is identified in over 90% of PXAs^7^; in the vast majority (>90%) of tumors showing *CDKN2A* HD, *CDKN2B* gene is also homozygously deleted.^8^ Currently, *CDKN2A* status can be assessed by different techniques, including fluorescence in situ hybridization (FISH), whole-genome copy number microarray-based assays, next-generation sequencing and, more recently, whole-genome DNA methylation arrays. However, conducting these tests for *CDKN2A*/*B* HD assessment frequently involves increased costs and thus restricted accessibility for many individuals and institutions.

*CDKN2A* HD results in loss of expression of p16, one of the gene products of *CDKN2A*. Consequently, p16 immunohistochemistry (IHC) has been used as a practical and cost-effective alternative for detecting this molecular alteration.^9^^,^^10^ As the methylthioadenosine phosphorylase (*MTAP*) gene is located in close genomic proximity (165 kb telomeric) to *CDKN2A* on 9p21 and deletions of this gene frequently co-occur with *CDKN2A* HD,^11^ loss of MTAP protein expression has also been proposed as a promising surrogate marker for *CDKN2A* HD detection in CNS and non-CNS tumors.^12–15^ The role of the combined use of p16 and MTAP IHC to predict *CDKN2A* status in PXA has not been fully investigated. Therefore, our study aimed to assess a cohort of patients with PXA and compare p16 and MTAP IHC with *CDKN2A*, *CDKN2B*, and *MTAP* deletion status by whole-genome copy number microarray-based analysis, and to determine if IHC is a reliable surrogate for assessing *CDKN2A* HD.

## METHODS

### Patient cohort

Thirty-eight patients diagnosed with PXA (CNS WHO grade 2, *n* = 23, 60.5%) or A-PXA (CNS WHO grade 3, *n* = 15, 39.5%) and available CMA data were included, 22 of which were included in previous studies.^7^^,^^16^ Following Institutional Review Boards (IRB) of Mayo Clinic approval, clinicopathologic and molecular information was retrospectively reviewed. Confirmation of the histologic diagnosis and CNS WHO grade was performed on all tumors by at least 2 neuropathologists (C.G., R.A.V., A.R., A.T.N., J.A.T.-L., M.A.V.), according to the 2021 WHO diagnostic criteria.^1^

### Immunohistochemistry

Immunohistochemistry was performed at Mayo Clinic on formalin-fixed, paraffin-embedded (FFPE) tissue using an automated immunostainer (Ventana Ultra platform). Primary antibodies were directed against MTAP and p16 (INK4a/CDKN2A) antigens. For MTAP, pretreatment with Cell Conditioning Solution (CC1, Ventana) for 32 min at 100 °C was performed, followed by primary antibody incubation for 32 min at 36 °C using MTAP mouse monoclonal (clone 2G4) Abnova (catalog H00004507-M01) antibody at a 1/1000 dilution with Dako Background Reducing diluent and Ventana Optiview DAB detection with hematoxylin II and bluing reagent. For p16, pretreatment with CC1 for 48 min at 100 °C was performed, followed by primary antibody incubation using CINtec Histology (p16 mouse antibody, clone E6H4, prediluted) Ventana/Roche (catalog number 725-4793) for 12 min at 36 °C and Ventana Optiview DAB detection with hematoxylin II and bluing reagent.

All PXAs were assessed and qualitatively scored blindly by 2 independent reviewers (C.G., M.A.V.), and p16 and MTAP immunostains were evaluated separately. Expression of p16 was scored by the presence (retained) or absence (lost) of nuclear staining, while MTAP was tallied by the presence (retained) or absence (lost) of cytoplasmic staining, with or without concurrent nuclear positivity. As MTAP nuclear staining has been shown to be inconsistent in previous studies,^12^^,^^17^^,^^18^ nuclear MTAP expression alone was not considered to render a score. Expression of these markers was considered as equivocal when there was no clear-cut loss of nuclear p16 positivity and when the absence of MTAP cytoplasmic staining was ambiguous, respectively.

### Genome-wide chromosomal copy number analysis

Genome-wide chromosomal microarray analysis (CMA) results available in 36 of 38 (94.7%) patients were retrospectively reviewed. In the remaining 2 (5.3%) cases, CMA testing was performed. In summary, DNA was extracted from 5-µm FFPE tissue sections using the QIAamp DNA FFPE Tissue Kit (Qiagen, Hilden, Germany).^19^ Genomic copy number losses and gains were identified with a molecular inversion probe array (OncoScan Copy Number Variation Assay, ThermoFisher Scientific, Waltham, MA, United States). The array has a 50- to 100-kb copy number resolution and covers frequent copy number changes across approximately 900 cancer genes, including detection of *CDKN2A*, *CDKN2B*, and *MTAP* homozygous/heterozygous deletions. Raw data were analyzed using the ChAS software (ThermoFisher Scientific). Whole chromosomes were counted as gained or lost when at least 90% of the probe signals from both chromosome arms were above or below the defined threshold.^20^ For acrocentric chromosomes, only q-arm changes were counted as whole chromosomal gains or losses. Chromosomal copy number changes including *CDKN2A*, *CDKN2B*, and *MTAP* deletion status were blindly assessed by independent reviewers (C.J.Z.M., R.B.J.).

### Statistical analysis

Data were summarized with frequencies and percentages or medians and ranges, as appropriate. Performance of p16 and MTAP expression to detect *CDKN2A* or *MTAP* HD was summarized with sensitivity (percentage with expression loss among those with HD present), specificity (percentage with expression retained among those with HD absent), and positive predictive value (PPV) (percentage with HD present among those with expression loss). The sensitivity for the combination of p16 and MTAP expression to detect *CDKN2A* HD was calculated as the percentage of HD patients who had loss of expression for either p16 or MTAP. All analyses were performed using R version 4.2.2.^21^

## RESULTS

### Clinicopathologic features

**Figure 1.**
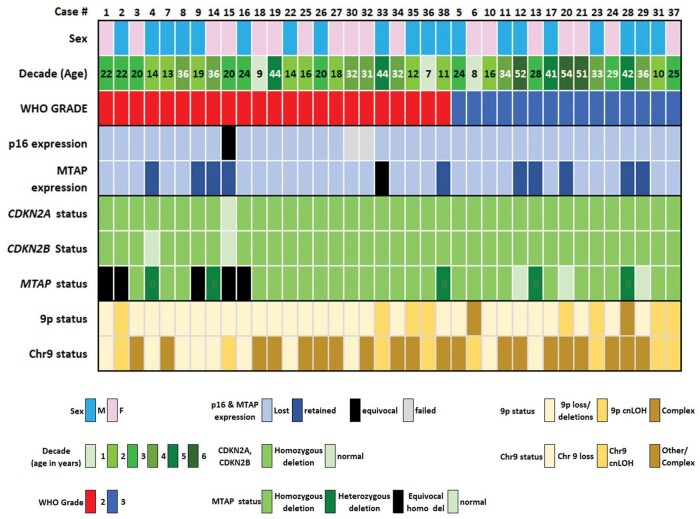
Summary of clinicopathologic and molecular features of the pleomorphic xanthoastrocytoma cohort. Expression of p16 and MTAP was qualitatively scored as lost (absent) or retained (present). *CDKN2A*, *CDKN2B*, and *MTAP* deletions, as well as whole chromosome 9 and 9p status, were evaluated by CMA analysis. MTAP = methylthioadenosine phosphorylase.

The clinicopathologic and molecular features of our PXA and A-PXA patient cohort are summarized in Figure 1. Twenty of 38 (52.6%) cases presented in males and 18 (47.4%) in females. Eleven (28.9%) PXAs occurred in pediatric patients (<18 years of age) and 27 (71.1%) in adults, with a median age at diagnosis of 24 years (range from 7 to 54 years). Most PXAs were located in the temporal lobe (*n* = 24, 63.2%), followed by parietal (*n* = 6, 15.8%) and frontal (*n* = 5, 13.1%) lobes, cerebellum (*n* = 2, 5.3%), and left cerebral hemisphere, not furtherly specified (*n* = 1, 2.6%). Twenty-three (60.5%) PXAs corresponded to CNS WHO grade 2 tumors, 20 of which were primary versus 3 recurrent PXAs. Fifteen (39.5%) PXAs were designated as CNS WHO grade 3 (A-PXA), 10 of them primary versus 5 recurrent tumors.

### Genomic features

**Figure 2.**
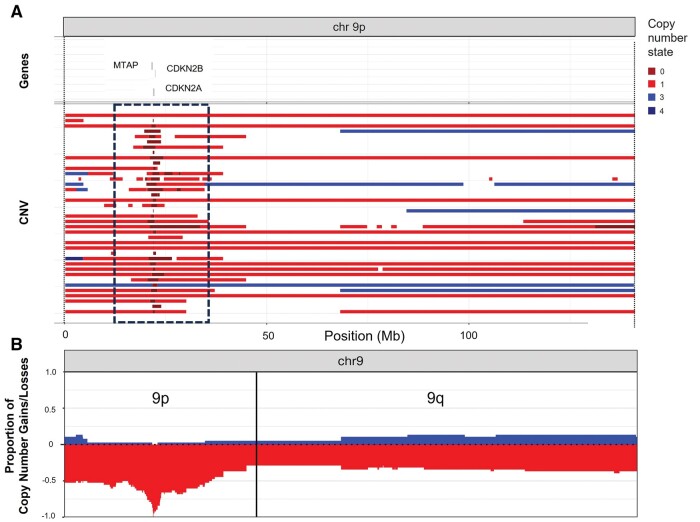
Summary of chromosome 9 copy number variation of the pleomorphic xanthoastrocytoma cohort. The areas with HD involving *CDKN2A*, *CDKN2B*, and/or *MTAP* on each case are represented in red wine color (framed). Regions of chromosome 9p losses and gains are illustrated in bright red and blue, respectively (A). Loss of chromosome 9p whole-arm loss was present in 18 of 38 cases, while whole-chromosome 9 loss was identified in 10 cases (B). MTAP, methylthioadenosine phosphorylase.

The summary of chromosome 9 copy number variation of this PXA cohort, including *CDKN2A*, *CDKN2B*, and *MTAP* status, is shown in Figures 1 and 2. Chromosomal copy number analysis demonstrated HD of *CDKN2A* in 37 of 38 (97.4%) cases, whereas *CDKN2B* HD was identified in 36 (94.7%) cases and *MTAP* was homozygously deleted in 25 (65.8%) PXAs (Figures 1 and 2A). Hemizygous *MTAP* loss was present in 5 of 38 (13.2%) cases, while in 5 (13.2%) cases *MTAP* deletion status could not be confidently interpreted (Figure 1), likely due to a combination of *MTAP* deletion sizes under the resolution of the CMA gene region probe coverage or lower tumor content (Figure S2). Chromosomal losses involving chromosome 9 included whole arm –9p (18/38, 47.4%), whole chromosome –9 (10/38, 26.3%), and 9p/chromosome 9 copy neutral loss of heterozygosity (cnLOH) (10/38, 26.3%) (Figures 1 and 2B). Additional chromosomal gains and losses were identified, including frequent (∼25% of cases) gain of chromosomes 5, 7, and 12 and loss of chromosomes 10, 13, and 22 (Figure S1). Three of 38 PXAs (1 CNS WHO grade 2 and 2 grade 3 tumors) harbored concurrent +7 and −10, a molecular finding that has been previously described in PXAs.^16^ Additional whole and partial chromosome gains and losses were observed at lower frequencies (<15%), including loss of 1p, 3p, chromosomes 8 and 11, 16q, 17p (including *TP53*), and 19q, and gains of 1q, chromosome 4, 17q (including *NF1*), and chromosomes 20 and 21 (Figure S1).

### Immunohistochemical features

**Figure 3.**
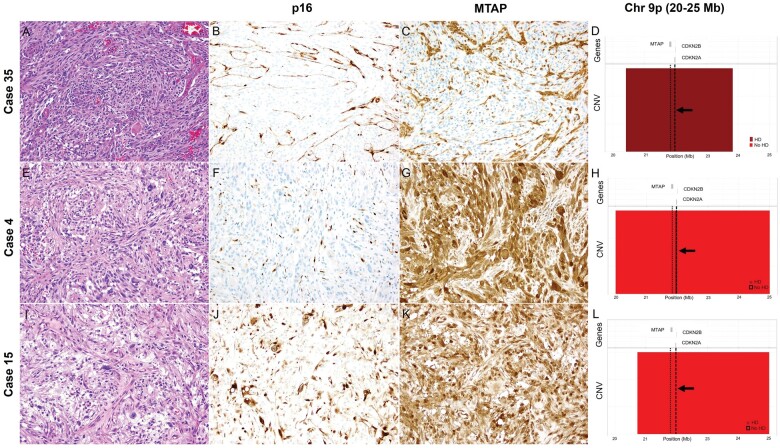
Comparison of p16 and MTAP IHC with *CDKN2A*, *CDKN2B*, and *MTAP* deletion status. Case 35 (A) illustrates a PXA with loss of p16 (B) and MTAP (C) immunoexpression. By CMA, HD of *CDKN2A*, *CDKN2B*, and *MTAP* were identified (D). In contrast, Case 4 (E) showed loss of p16 (F) with retained MTAP expression (G). CMA demonstrated HD of *CDKN2A*, but not of *CDKN2B* or *MTAP* (H). Case 15 (I) corresponds to the single PXA in which p16 expression was equivocal (J), while MTAP expression was retained (K). Interestingly, this was also the single case in which no deletions of *CDKN2A*, *CDKN2B*, or *MTAP* were identified by CMA (L). MTAP = methylthioadenosine phosphorylase; IHC = immunohistochemistry; PXA = pleomorphic xanthoastrocytoma.

Loss of p16 expression was identified in 35 of 38 (92.1%) PXAs, all (35 of 35) with the presence of *CDKN2A* HD (Figure 3A, B, D-F, and H). Two of 38 (5.3%) cases failed to stain with this marker, and in a single (2.6%) PXA the expression was interpreted as equivocal (Figure 3I and J) (sensitivity = 94.6%; PPV=100%). As nearly all samples had *CDKN2A* HD and none had retained p16 expression, specificity or negative predictive value (NPV) for p16 in predicting *CDKN2A* status could not be determined in this cohort.

Evaluation of MTAP was successful in all 38 (100%) PXAs. Expression of MTAP was lost in 27 of 38 (71.1%) cases, all of which demonstrated HD of *CDKN2A* and *CDKN2B* by CMA (PPV = 100%) (Figure 3C and D). A subset (11 of 38) of PXAs, however, showed retained (10, 26.3%) (Figure 3G and H) and equivocal (1, 2.6%) MTAP staining, respectively, while harboring *CDKN2A* HD. Among the 37 with HD of *CDKN2A*, 27 had lost MTAP expression (sensitivity = 73%). Among the 25 (of 38) cases harboring *MTAP* HD, 24 showed loss of MTAP expression, while in one case MTAP expression was equivocal. All 8 cases in which *MTAP* HD was absent had retained MTAP expression (sensitivity = 96.0%, specificity = 100%).

Remarkably, the 2 cases in which p16 staining failed to demonstrate loss of MTAP expression and CMA confirmed the presence of *CDKN2A*, *CDKN2B*, and *MTAP* HD, resulting in a combined sensitivity (loss of p16 and/or MTAP expression) to detect *CDKN2A* HD of 100%. Of note, the isolated case (Case 15) with equivocal p16 staining was the only PXA in which no HD of *CDKN2A* or *CDKN2B* was detected by CMA. This case also showed retained MTAP expression and no definitive *MTAP* deletion by CMA (Figure 3I-L).

**Figure 4.**
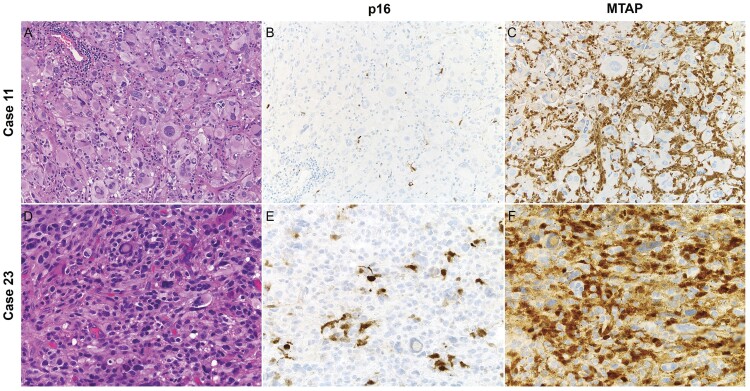
Difficulty to assess for p16 and MTAP IHC in a subset of pleomorphic xanthoastrocytomas. Case 11 (A) showed scant p16 staining in blood vessels and non-neoplastic cells (B), making the evaluation problematic but ultimately interpreted as lost given the lack of expression within the tumor and retained staining in background cells. The expression of MTAP in this case was lost with appropriate internal control (C). On the other hand, Case 23 (D) showed loss of p16 expression with good internal control (E), but strong background staining of MTAP was noted (F), making the interpretation also challenging. Given the absence of cytoplasmic staining in tumor cells, MTAP expression was considered as lost. MTAP = methylthioadenosine phosphorylase; IHC = immunohistochemistry.

It is worth mentioning that p16 IHC overall generally showed less staining in background blood vessels and non-neoplastic cells (positive internal controls). This was more prominent within the brain parenchyma compared to the leptomeninges, potentially making the interpretation challenging in a subset of cases due to the paucity of a positive internal control (Figure 4A-C). MTAP, on the other hand, demonstrated in general high background staining, including in macrophages, being quite prominent in a subset of cases and making the interpretation also challenging (Figure 4D-F).

## DISCUSSION

*CDKN2A* gene inactivation is a common event in cancer. This often occurs by HD but alternative mechanisms like point mutations and gene promoter hypermethylation can also induce *CDKN2A* inactivation.^22^^,^^23^ High rates of *CDKN2A* HD have been identified in several neoplasms, including pleural mesothelioma, pancreatic ductal adenocarcinoma, malignant peripheral nerve sheath tumor, cutaneous melanoma, and colorectal carcinoma, among others.^22^^,^^24^^,^^25^ Within CNS tumors, *CDKN2A* HD is a hallmark of PXA,^7^ and more recently it has been recognized as an adverse prognostic factor in IDH-mutant diffuse gliomas, pediatric low-grade glial/glioneuronal tumors, supratentorial ependymomas, and meningiomas.^26–31^ Therefore, the detection of *CDKN2A* HD for diagnostic and prognostic purposes has become increasingly relevant. Considering the limited accessibility of molecular testing to selected centers and the substantial rise in costs, IHC may prove to be a valuable alternative for evaluating this genetic alteration.

Although p16 IHC does not provide information about the mechanism of *CDKN2A* inactivation, it does correlate with the status of *CDKN2A*.^25^ For that reason, p16 has been used as a surrogate marker to assess for *CDKN2A* HD in different tumors.^9^^,^^10^ Given the chromosomal proximity of *MTAP* to the *CDKN2A* locus and the high frequency of concurrent deletions,^11^ MTAP IHC loss has been suggested as an alternative to evaluate for *CDKN2A* HD in different CNS and non-CNS tumors, including a recent study in PXA.^12–15^^,^^17^ Additionally, recent studies have shown early clinical success of protein arginine methyltransferase 5 (PRMT5) inhibitors to treat patients with *CDKN2A/MTAP*-deleted tumors, which adds to the relevance of assessing for these molecular abnormalities.^32^^,^^33^ Although the utility of combined p16 and MTAP IHC as a substitute for *CDKN2A* molecular testing has been previously reported in several cancers, including esophageal noninvasive precursor lesions, pancreatic neoplasia, lung cancer, pleural mesothelioma, and diffuse and circumscribed gliomas,^24^^,^^25^^,^^34–36^ to the best of our knowledge this has not been explored in PXA.

Here, we have compared the utility of p16 and MTAP IHC as a surrogate marker to assess for *CDKN2A* HD in a cohort of 38 patients with PXA and CMA data. Homozygous deletion of *CDKN2A* and *CDKN2B* was present in most cases (37/38, 97.4% and 36/38, 94.7%, respectively), while concurrent HD of *MTAP* was detected in 25/37 (67.6%) PXAs. p16 alone highly correlated with *CDKN2A* HD (sensitivity = 94.6%). Although it is not possible to determine the specificity and NPV for p16 to predict *CDKN2A* status in our cohort as nearly all samples harbored *CDKN2A* HD and none had retained p16 expression, previous studies evaluating a variety of circumscribed and infiltrating glial and non-glial CNS neoplasms have shown that p16 IHC has both a strong sensitivity and specificity (>90%-100%) and high NPV (100%) to detect *CDKN2A* deletion.^9^^,^^10^^,^^37^ Additionally, the few formerly reported studies concurrently assessing for p16 and MTAP IHC in circumscribed and adult-type diffuse gliomas^28^^,^^38^^,^^39^ have shown variable results but, overall, a high sensitivity (from 87% to >90% and up to 100% for p16 and MTAP, respectively) and specificity (up to 89% and 97% for p16 and MTAP, respectively) of these markers to detect HD of *CDKN2A/B*. In our PXA cohort, the combination of p16 and MTAP IHC correctly detected this molecular alteration, reaching a sensitivity of 100%. Notably, even though MTAP IHC alone demonstrated a high sensitivity and specificity in detecting *MTAP* HD (96% and 100%, respectively), it showed a low sensitivity (73%) to discern *CDKN2A* HD in this PXA cohort, which is explained by the absence of *MTAP* HD on those cases. Although Lou et al^13^ found a higher correlation between MTAP expression and *CDKN2A* status, this could be attributed to the assessment of *CDKN2A* HD through FISH, which has a lower rate of *CDKN2A* HD detection compared to array-based assays.

An important consideration when interpreting these markers is their IHC staining pattern. Significant paucity of internal controls for p16 can make the evaluation challenging in some cases, especially within the brain parenchyma (Figure 4B). In contrast, even though MTAP demonstrated good internal controls overall, its interpretation in a subset of cases can be difficult due to significant staining of endothelial cells, tumor-associated macrophages, and non-neoplastic glial cells, more prominently seen in the brain parenchyma than in the leptomeninges (Figure 4F).

In conclusion, our findings indicate that loss of p16 expression showed a good correlation with *CDKN2A* status, while MTAP alone had a lower sensitivity in detecting HD of *CDKN2A* in PXA. Nonetheless, the use of combined p16 and MTAP IHC correctly predicted this molecular abnormality and, therefore, this could be a reliable method to assess for *CDKN2A* HD in PXA when molecular testing is not available.

## Supplementary Material

nlae076_Supplementary_Data
