# Supplementary figures and images for: Comparing loss of p16 and MTAP expression in detecting CDKN2A homozygous deletion in pleomorphic xanthoastrocytoma
Source: J Neuropathol Exp Neurol. 2024 Jul 23;83(12):1003–9. doi: 10.1093/jnen/nlae076 (PMC11576554; doi:10.1093/jnen/nlae076)

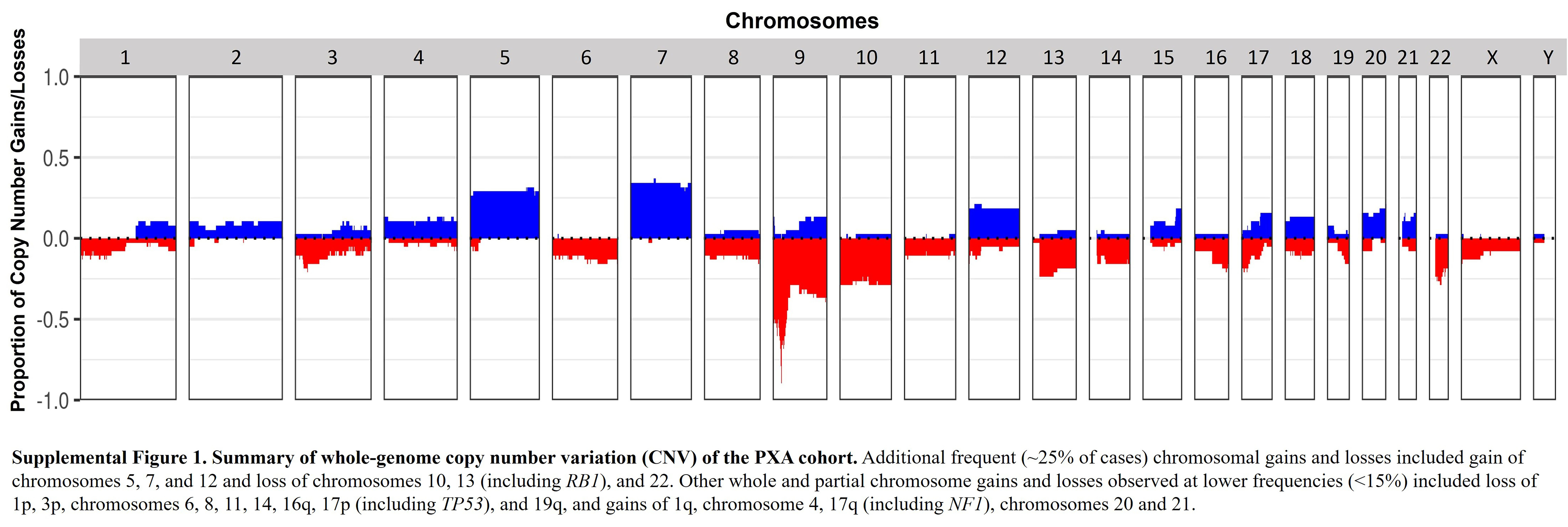

Supplement: nlae076_Supplementary_Data [file nlae076_supplementary_data.zip › nlae076_Supplementary_Data/Supplemental figure 1 (modified).jpg]

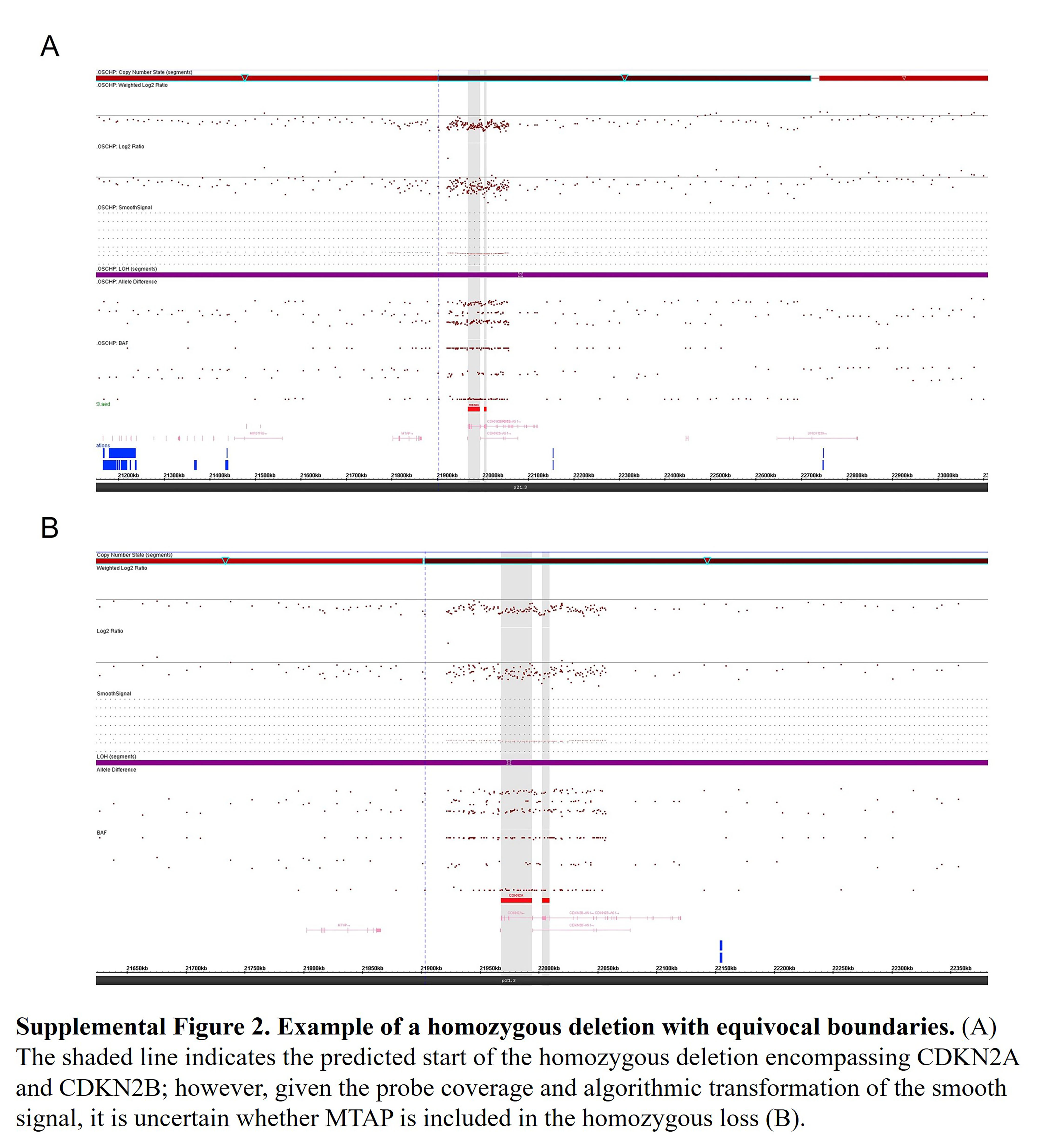

Supplement: nlae076_Supplementary_Data [file nlae076_supplementary_data.zip › nlae076_Supplementary_Data/Supplemental figure 2 (modified).jpg]
